# Confidence degree and skill development in undergraduate medical students using male urogenital training simulators

**DOI:** 10.1590/0100-6991e-20243593-en

**Published:** 2023-12-20

**Authors:** Malcom Jones Krummenauer Brigo, Maria Cecília Da Lozzo Garbelini, Izabel Cristina Meister Martins Coelho

**Affiliations:** 1 - Universidade Estatual do Oeste do Paraná, CCMF - Cascavel - PR - Brasil; 2 - Faculdades Pequeno Príncipe, Departamento de Pesquisa e Pós Graduação - Curitiba - PR - Brasil

**Keywords:** Urogenital System, Competency-Based Education, Simulation Training, Treinamento por Simulação, Sistema Urogenital, Educação Baseada em Competências

## Abstract

**Introduction:**

medical training should provide the future professional, in addition to theoretical knowledge, general and specific skills. In urology, urogenital training simulators have been presented as an ally in improving the degree of confidence and development of competencies for undergraduate medical students.

**Methods:**

exploratory descriptive research with a quantitative approach, of an experimental nature, of the randomized controlled type with cross-sectional cut. Conducted with the students of the 4^th^ year of medicine of a Higher Education Institution in the West of Paraná.

**Results:**

91 students attended a theoretical class with a complete explanation of the activities to be performed and answered the initial questionnaire about the degree of confidence to perform tasks in three stations with male urogenital training simulators (prostatic touch, bladder catheterization and scrotal evaluation). Of these, 45 received guidance and training with the simulators prior to the stations, while 46 should demonstrate skills directly in the three stations, mimicking patient care, only with information from the theoretical classes. The students who received previous guidance with the simulators had their scores in the development of competence higher. And, when they repeated the questionnaire about the degree of confidence to demonstrate skills with the mannequins, there was a higher degree of confidence in performing the tasks, except for the execution of a task considered more difficult.

**Conclusion:**

there was an improvement in the degree of confidence and in the development of competencies of undergraduate medical students with the orientations in the male urogenital training simulators.

## RESUMO

**Introdução:**

a formação médica deve fornecer ao futuro profissional, além de conhecimentos teóricos, habilidades gerais e específicas. Na urologia, os simuladores de treinamento urogenital, têm se apresentado como aliado na melhora do grau de confiança e desenvolvimento de competências para estudantes de graduação em medicina.

**Métodos:**

pesquisa exploratória descritiva, com abordagem quantitativa, de natureza experimental, do tipo randomizado controlado com recorte transversal. Realizada com os estudantes do 4º ano de medicina de uma Instituição de Ensino Superior no Oeste do Paraná.

**Resultados:**

91 estudantes assistiram a uma aula teórica com explanação completa das atividades a serem realizadas, e responderam ao questionário inicial sobre o grau de confiança para executar tarefas em três estações com simuladores de treinamento urogenital masculino (toque prostático, cateterismo vesical e avaliação escrotal). Destes, 45 receberam orientações e treinamento junto aos simuladores previamente as estações, enquanto 46 deveriam demonstrar habilidades diretamente nas três estações, mimetizando o atendimento ao paciente, apenas com informações das aulas teóricas. Os estudantes que receberam orientação prévia junto aos simuladores, tiveram seus escores no desenvolvimento de competência mais elevados. E, ao repetirem o questionário sobre o grau de confiança para demonstrar habilidades junto aos manequins, houve maior grau de confiança em executar as tarefas, com exceção da execução de uma tarefa considerada mais difícil.

**Conclusão:**

houve melhora no grau de confiança e no desenvolvimento de competências dos estudantes de graduação em medicina com as orientações nos simuladores de treinamento urogenital masculino.

## INTRODUCTION

In Brazil, the Ministry of Education established the National Curricular Guidelines (DCN) for the Undergraduate Medicine Course in 2001, which represented an important change in the curricular organizations of Higher Education Institutions (HEIs). The DCN, which were updated in 2014, established the principles, foundations, and purposes of medical training, guiding that courses must train generalist, humanist, critical, and reflective professionals, who work in health promotion, prevention, recovery, and rehabilitation actions^1^.

The DCN establish that the contents of undergraduate medical courses must be related to the entire health-disease process, providing comprehensive care actions in medicine, requiring graduates to have a perfect articulation between knowledge, skills, and attitudes for professional practice^1^.

In urology, in addition to theoretical training, students must well develop the practical part of examinations and procedures of the urogenital tract (UGT), and usually causes great anxiety in them^2^. The training students usually receive in traditional teaching methods is often known as: “see one, do one, teach one”, which can lead to a deficiency in learning, as well as being a source of great stress and frustration^3^.

Concern about these forms of teaching, which can cause learning deficits, has led to the search for new instructional modalities that systematize teaching, through the development of techniques and skills, improving the way of learning and reducing student stress.

In this scenario, simulators have represented a great ally, evolving and attracting students from the most varied areas of knowledge. Simulations aim to provide information with real-life characteristics, allowing participation to experience situations close to those undergone in everyday life.

Studies have demonstrated that systematic training with simulators and mannequins, prior to direct contact with the patient plays a significant role in developing students’ skills^3^ ^,^ ^4^, in addition to being feasible and implemented without major disruption, improving the physician curriculum^5^.

Studies show that simulation can be applied both to teach and to evaluate, since considering simulation a practical activity, it is possible to evaluate both knowledge and skill^6^.

Another key factor that suggests the need for systematization in UGT learning is the considerable number of studies showing that there is a failure in this training in Urology, which has led new doctors to conduct inaccurate diagnoses and cause iatrogenic injuries, even in procedures considered simple^7^. 

Thus, the question was: Is it possible to improve the level of confidence and development of skills through the systematization of teaching related to the urogenital tract using simulators for undergraduate medical students?

## METHODS

The research is exploratory, descriptive, with a quantitative approach, experimental in nature, randomized, controlled, and with a cross-sectional approach^8^.

We conducted it at a higher education institution (HEI) in western Paraná. Undergraduate medical students who were attending the discipline of Urology and who were 18 years of age or older participated in the research.

We excluded from the research the students who participated in academic Urology leagues, those who had previously completed internships in the specialty, those who did not want to undergo activities using mannequins, or who did not complete the questionnaires.

The project was previously sent to an Ethics in Research Committee (CEP), in accordance with Resolution 466/12 of the National Health Council9 and was approved under opinion 4,698,241.

### Description of the research steps

Stage 1: Participants received information about the research and after signing the Free and Informed Consent Form (TCLE), they attended the theoretical class, with details of the activities that they were expected to demonstrate skills in.

Stage 2: Students responded to a questionnaire (Q1) with sociodemographic information, and about their previous knowledge about their activities in Urology, seeking to identify the current level of information and training about the activities that were developed.

Step 3: Pre-test. The students responded to a questionnaire (Q2) with a Likert Scale (attached) about the degree of understanding of the activities exposed in the class they had just participated in before contact with the stations with mannequins.

Step 4: The students were divided into two groups, G1 (Intervention) and G2 (Control). G1, had the information obtained in the expository class and a post-training session with an advisor, who demonstrated the correct way to carry out the procedures with the model mannequins; later, under the supervision of two evaluators, they provided care to the simulated patient at the stations of male bladder catheterization, prostate assessment, and male perineal assessment, all with the assessment of the observed competencies highlighted through the checklist. The G2 group should count only on the information obtained in the expository class, and under the supervision of two evaluators, demonstrate understanding in simulated patient care with mannequins, divided into the same three stations, also undergoing the checklist assessment of competence.

We divided the students into groups by random sampling and the evaluators did not receive information about the student’s previous situation, that is, we attempted to create a double-blind model to limit the evaluators’ knowledge regarding the students’ training level. This reduces the possibility of inducing a response from evaluators, as recommended in randomized clinical trials

### Description of stations

In the expository class, all students received instructions on how to conduct the procedures they would find in the three stations to be studied:

Station 1: Male bladder catheterization on the simulated mannequin

Station 2: Prostate exam on the simulated prostate mannequin

Station 3: Male perineal examination on the scrotal mannequin

We randomly divided them into two groups, odd-numbers students receiving training next to an instructor’s mannequin, and even-numbers students, only with the instructions of the expository class, without an instructor.

At each station, the routine was the same, and the student had three minutes to conduct the activity with the mannequin:

1. Identify the medical record next to the manikin (by looking or picking the medical record up and reading it).
2. Introduce yourself to the patient (mannequin).
3. Identify the manikin as a patient by name.
4. Explain the procedure to the mannequin patient.
5. Put both gloves on correctly.
6. Perform local asepsis with gauze.
7. Conduct the station procedure.
  - Bladder catheterization: insert the catheter, observe the urine output - the mannequin emits a beeping sound when the catheter reaches the bladder and urine comes out (in this case, serum); dispose of the catheter and glove in the correct place.
  - In the prostate examination: perform the examination and identify the prostate pathology (in this case, a single prostatic nodule); dispose of the glove in the correct place.
  - In the perineal examination: palpate the silicone scrotum, identify the testicular nodule; dispose of the glove in the correct place.
8. Assessment of material disposal.

At all stations, students were evaluated by the researcher, the invited Urology professor, and a nursing technician who performs surgical instrumentation, with the notes recorded on the single form by mutual agreement or by majority.

After developing the activities at the stations with mannequins, the students who received training responded to a new questionnaire about their level of confidence and their ability to develop skills.

## RESULTS

The population available for the study had 98 students who, after completing the requirements to participate in the research, resulted in a sample of 91, aged between 19 and 40 years. Table 1 presents the descriptive results of the average scores obtained by students at each station, divided between those who underwent training with the advisor (G1) and those who did not (G2).

**Table 1:** Average scores obtained by students with and without prior training according to checklist in each of the three scenarios.

	With Training		No Training	
	Average	SD	Average	SD
Station 1	8,085	1,736	6,065	1,621
Station 2	6,319	1,401	4,783	1,350
Station 3	6,340	1,403	5,067	1,356
			(p<0.001)	

Station 1 was the easiest for students to perform in both groups. Station 3 was the second in order of difficulty and station 2 was the third. These data indicate that, regardless of receiving training, the passage of a urinary catheter was considered the easiest procedure to perform, while the prostate exam was the most complex.

[Fig f1]

**Figure 1: f1:**
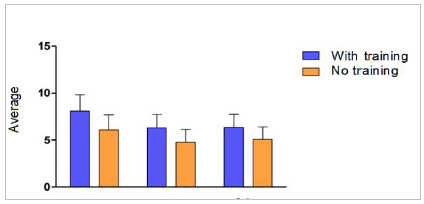
Comparative data between types of training and scenarios (C.1, C.2, C.3) evaluated as per the checklist.

This result is confirmed by Two-Way Analysis of Variance (ANOVA). The column factor (Training level) was statistically different in the two groups (p<0.001, F=79.87, dF=1). Likewise, the line factor (different stations) was statistically distinct (p<0.001, F=28.95, dF=2), demonstrating that each station presents different results and can be categorized in order of difficulty.

Although the scores of students who received training were higher, their assessment using a checklist showed variations within each station.

The degree of difficulty is assessed by logistic regression of the number of students who do not reach a certain item. Thus, question number 4 is one where 57.4% of students did not comply with what was proposed. This item refers to “explaining the procedure to the patient”.

Considering the “simplified” aspect of this operation, we observed that the student believes that it is of little importance to report the procedure to the patient and would only do so with prior training. This phenomenon is reinforced in the group without training, where the number of students who did not complete the task (76.1) is 18.7% higher.

For practical purposes, each 0.2 points corresponds to a level of difficulty. Thus, 0.00 0.199 corresponds to exceedingly difficult items, followed by 0.200 0.399 (difficult), 0.400 0.599 (medium), 0.600 0.799 (easy), and 0.800 1.000 (very easy).

We observed that the teacher can safely evaluate the student who developed the proposed skill when questions of medium or high difficulty are reached, unlike the very easy ones (above 0.8 in both groups), where these questions do not demonstrate technical ability themselves, since both students with and without training fulfill these items perfectly. Those are passing the catheter correctly, identifying the medical record, putting on gloves correctly, and draining the urine.

Two-Way Analysis of Variance (ANOVA) confirms this data. The column factor (Training level) was statistically different in the two groups (p<0.01, F=66.98, dF=1). Likewise, the line factor (different stations) was statistically distinct (p<0.01, F=43.83, dF=8), demonstrating that each station presents different results and can be categorized in order of difficulty (Table 2). 

**Tabela 2:** Level of difficulty of the questions obtained by the item response theory of three logistical parameters ordered in increasing order of ease for scenario 1.

	Scenario 1						
	With Training			No Training			
Item	Difficulty	SD	DI	Item	Difficulty	SD	DI
4	0.426	0.494	0.337	4	0.239	0.427	0.204
9	0.532	0.499	0.254	9	0.152	0.359	0.102
	Scenario 1						
	With Training			No Training			
Item	Difficulty	SD	DI	Item	Difficulty	SD	DI
10	0.660	0.474	0.164	10	0.261	0.439	0.250
3	0.787	0.409	0.407	3	0.413	0.492	0.411
6	0.872	0.334	0.288	6	0.630	0.483	-0.102
2	0.915	0.279	0.517	2	0.609	0.488	0.257
7	0.957	0.202	0.537	7	0.957	0.204	0.219
1	0.979	0.144	0.639	1	0.935	0.247	0.434
5	0.979	0.144	0.639	5	0.913	0.282	0.232
8	0.979	0.144	0.639	8	0.957	0.204	0.436

After Bonferroni post-test correction, items 9, 10, 3, 6, and 2 were considered distinct from each other, demonstrating that these questions tend to better differentiate students who have the competence required in this scenario.

### Comparative analysis of pre-test questionnaires

After the expository class, all students answered a questionnaire (Q2a) to assess their understanding of the theoretical aspects of the topic, as well as their confidence in performing the required techniques.

The 45 students who received prior training with the teacher (Q2b) answered the questionnaire a second time, with the same questions, to check their level of understanding and confidence after completing practical training.

**Figure 2:**
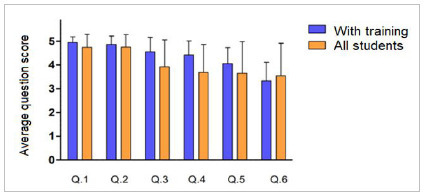
Comparative analysis between the questionnaires on the level of understanding and confidence, answered by all students and only by those who underwent practical training.

From Figure 2, it is possible to note that the average score obtained by students with training is higher in the first five questions, before training with a teacher. In the sixth question, which refers to the student’s confidence in teaching a patient to perform bladder catheterization at home, the score decreased, suggesting that the need to perform a more difficult task makes them aware of the degree of their difficulty and their confidence decreases.

### Odds ratio calculation

To calculate the odds ratios, an average degree of difficulty was listed, that is, the relationship between students who achieved a minimum of 50% correct answers in each scenario, as shown in Table 3. In this model, we did not take into account the difficulty of the scenario, so that it was possible to compare the ratios of the three scenarios with each other.

**Table 3:** Calculation of odds ratios in the three scenarios discussed.

	Scenario 1	Scenario 2	Scenario 3
Odds ratio	1.48	1.80	2.0
95% confidence interval	0.64 - 3.40	0.77 - 4.23	0.84 - 4.72

From the data obtained, students who went through the training were, respectively, 1.48, 1.80, and 2.0 times more likely to obtain higher scores than those who did not go through the training.

## DISCUSSION

The present research addressed the development of skills in undergraduate medical students using urogenital training mannequins and contributed to the analysis of students performing procedures after theoretical training and in conjunction with training on mannequins.

Simulation has emerged as a tool that can provide efficient cost-benefit in terms of training time and safety, and even more so, patient safety. In 2008, the Surgery Residency Review Committee decreed that all training programs within the United States must “include simulation and skills laboratories”^12^.

As shown in Table 1, trained students found it easy to carry out the practice, which corroborates the findings by Motta^10^, which describes that the development of a broad educational process, covering different skills, allows the teacher to identify the positive and negative aspects of different students, making it possible to establish recovery strategies in training, using different simulation modalities.

This fact points to the importance of training with mannequins as a strategy in the training of students, using several types of simulators as a way of consolidating the theoretical knowledge acquired.

A randomized study^2^ showed the benefits of classes supported on mannequins versus traditional studies in reducing students’ anxiety when conducting assessments of patients’ urogenital tracts at the bedside compared to students who only took theoretical classes. The work of Rodrígues-Díez11 demonstrated that training with urology simulators for bladder catheterization and rectal examination improved student confidence in these skills. 

Regarding the impact of simulators on student confidence, it can be stated that with the inclusion of simulators in training programs, the degree of student confidence, as well as skills acquired, was significant.

Thus, we noticed that, as for competence, students who underwent training have a higher average score compared with those who have not received prior training, regardless of the station. Therefore, the level of students’ training affects their performance in the assessment.

Furthermore, it can be inferred that practice with mannequins brings a new meaning to students, because, as the practical part relates with the expository class, that is, their previous theoretical knowledge, mannequins bring a new structure of information, making students solidify their learning, improving their confidence.

Therefore, the work developed, in agreement with several authors mentioned above, demonstrated the need for changes in the way of teaching, with activities that use mannequins to simulate the urogenital tract by undergraduate medical students, to improve their level of confidence; and, through the systematization of methods, acquire new skills in urology.

It can be stated, after analyzing the results, that the development of medical skills regarding urology learning is positively affected the closer the students are to the content. This includes theoretical approaches, as well as practical or clinical applications of the content studied.

Meanwhile, the activities developed solely based on the theoretical class led to a deficit in completing tasks with the mannequins, causing greater stress and anxiety in students.

Thus, we suggest that through the implementation of the use of urinary tract simulation mannequins in the urology discipline, new work can be carried out to improve the method, aiming for student comfort, patient safety, and a more balanced medical training between theory in the classroom and clinical practice in contact with the patient. In this sense, we reinforce the need to increase the sample size.

## CONCLUSIONS

The improvement in understanding and execution of procedures by undergraduate medical students after theoretical and practical training with mannequins reinforces that the search for new teaching modalities, such as the use of urogenital training simulators, is fundamental in the evolution of learning and in the degree of confidence.

The results demonstrated a change in the students’ aptitude and development of competence to conduct semiology of the urogenital tract and procedures such as male bladder catheterization, rectal examination, and male perineal examination after training with simulators.

## References

[1] Brasil. Ministério da Educação. Conselho Nacional de Educação. Câmara de Educação Superior (2014). Resolução CNE/CES n.o 3, de 20 de junho de 2014. Institui diretrizes curriculares nacionais do curso de graduação em Medicina e dá outras providências.

[2] Pugh CM (2012). Use of Mannequin-Based Simulation to Decrease Student Anxiety Prior to Interacting With Male Teaching Associates. Teach Learn Med.

[3] Kaplan AG (2009). Preliminary Evaluation of a Genitourinary Skills Training Curriculum for Medical Students. J Urol.

[4] Naylor RA (2009). Can medical students achieve skills proficiency through simulation training. Am J Surg.

[5] Dayal AK (2009). Simulation Training Improves Medical Students' Learning Experiences When Performing Real Vaginal Deliveries. Simul Healthc.

[6] Vozenilek JS, Huff J, Reznek M, Gordon J (2004). See one, do one, teach one advanced technology in medical education. Acad Emerg Med.

[7] Davis NF (2016). Incidence, Cost, Complications and Clinical Outcomes of Iatrogenic Urethral Catheterization Injuries A Prospective Multi-Institutional Study. J Urol.

[8] Gil AC (1997). Metodologia do ensino superior.

[9] Brasil (2009). Ministério da Educação. Secretaria de Ensino Superior. Ministério da Saúde. Secretaria da Gestão do Trabalho e Educação na Saúde. Matriz de correspondência curricular para fins de revalidação de diplomas de médico obtidos no exterior / Ministério da Educação, Ministério da Saúde.

[10] Motta EV, Baracat EC (2018). Treinamento de habilidades cirúrgicas para estudantes de medicina - papel da simulação / Surgical skills training for medical students - role of simulation. Rev Med (São Paulo).

[11] Rodríguez-Díez MC (2014). La simulación mejora la confianza de los estudiantes para adquirir competencias en urología. Actas urol. esp.

[12] Brewin J, Ahmed K, Challacombe B (2014). An update and review of simulation in urological training. Int J Surg.

